# Foliar application of putrescine, salicylic acid, and ascorbic acid mitigates frost stress damage in *Vitis vinifera* cv. ‘Giziluzum’

**DOI:** 10.1186/s12870-023-04126-w

**Published:** 2023-03-10

**Authors:** Ilnaz Jalili, Ali Ebadi, Mohammad Ali Askari, Sepideh KalatehJari, Mohammad Ali Aazami

**Affiliations:** 1 Department of Horticulture and Agronomy, Science and Research Branch, Islamic Azad University, Tehran, Iran; 2 Department of Horticulture, College of Agriculture, University of Tehran, Tehran, Iran; 3 Department of Horticulture, Faculty of Agriculture, University of Maragheh, Maragheh, Iran

**Keywords:** *Vitis vinifera*, Reduction of ascorbate, Hydrogen peroxidase, Catalase

## Abstract

**Background:**

Cold stress is an effective factor in reducing production and injuring fruit trees. Various materials, such as salicylic acid, ascorbic acid, and putrescine, are used to alleviate the damage of abiotic stress.

**Results:**

The effect of different treatments of putrescine, salicylic acid, and ascorbic acid on alleviating the damage of frost stress (− 3 °C) to grapes ‘Giziluzum’ was investigated. Frost stress increased the amount of H_2_O_2_, MDA, proline, and MSI. On the other hand, it decreased the concentration of chlorophyll and carotenoids in the leaves. Putrescine, salicylic acid and ascorbic acid significantly increased the activities of catalase, guaiacol peroxidase, ascorbate peroxidase, and superoxide dismutase under frost stress. Following frost stress, the grapes treated with putrescine, salicylic acid, and ascorbic acid showed higher levels of DHA, AsA, and AsA/DHA than the untreated grapes. Our results showed that the treatment with ascorbic acid outperformed the other treatments in adjusting frost stress damages.

**Conclusion:**

The use of compounds, such as ascorbic ac id, salicylic acid, and putrescine, modulates the effects of frost stress, thereby increasing the antioxidant defense system of cells, reducing its damage, and stabilizing stable cell conditions, so it can be used to reduce frost damage to different grape cultivars.

## Background

Plants are constantly exposed to various environmental stresses [1]. Frost stress and cold injury is one of the main environmental factors that limit the agricultural productivity and geographical distribution of many plant species [2–8]. Low temperatures may reduce biosynthetic activity and membrane fluidity, inhibit the normal functioning of physiological and biochemical processes, impair metabolic function, and in some cases, cause permanent damage or death of plants [9–13]. When the membrane is subjected to a temperature below the required level, it transforms from a liquid to a gel state which, in turn, disrupts the membrane’s dynamism and functioning. The plasma membrane is a highly organized system that plays an important role in the relationship between the cell and the extracellular environment. In general, the result of frost stress is the loss of the membrane’s health and the leakage of salts [14]. In the process of responding to frost stress, a set of osmotic-regulating metabolites including soluble sugars and compounds, such as proline and glycine betaine, can reduce osmotic stress, expansion, water uptake, and the metabolic activity of plant cells [15]. Frost stress reduces plant growth and thus plant yield because there will be fewer carbohydrates available for crop production [16].

Photosynthesis is the most important and vital process in plants [17]. Frost stress may reduce the photosynthetic efficiency of plants by reducing the turnover of D1 protein at the reaction center of photosystem II or by reducing photosynthetic pigments such as chlorophyll [18]. Carotenoids may also waste extra energy through the xanthophyll cycle and protect the reaction center of photosystem II [19]. In citrus, many examples have been shown in different citrus species that photosynthesis and photosystems are damaged in the presence of low temperatures. Photosynthesis is reduced, photoinhibition occurs, and PSII stops functioning to reduce Fv/Fm values [20, 21]. Rice plants can adapt to frost stress by increasing the accumulation of antioxidants and antioxidant enzymes through neutralizing reactive oxygen species (ROS) [22]. To reduce the negative effects of ROS, plants use enzymatic antioxidants, including superoxide dismutase, ascorbate peroxidase, catalase, guaiacol peroxidase, and non-enzymatic antioxidants such as ascorbate acid and glutathione [23]. Ascorbate acid and glutathione play an important role in the antioxidant process of plants [24], and their concentrations increase under biological and non-biological stresses in plants [25, 26].

Polyamines are growth regulators of low-molecular weight plants found in aliphatic amines form. Common polyamines in plants are putrescine, spermidine, and spermine [27]. Putrescine, as one of the major polyamines, has important functions in the growth and differentiation of plants, as well as their responses to stresses [28, 29]. Exogenous applications of Polyamines modulated drought responses in wheat through accumulating osmolytes, regulating metabolism, and increasing free Polyamines [30]. Li et al. [31] proved that putrescine reduced drought-induced ROS accumulation in maize by increasing the activity of antioxidant enzymes. Different concentrations of putrescine and proline reduce the production of hydrogen peroxide and improve the antioxidant activity of leaves under frost stress [32]. Salicylic acid emerges as a key plant defense hormone with various vital roles in plant safety and is involved in systemic-acquired resistance in several plant tissues, including fruits [33]. The role of salicylic acid in frost tolerance is not fully understood. However, currently, some studies show that this acid can prevent oxidative damage caused by frost stress through regulating the antioxidant system [34, 35]. The application of salicylic acid increased the tolerance of cucumber seedlings to low-temperature stress [36]. Pretreatment of frost -sensitive banana plants with a 0.5 mM salicylic acid solution increased frost tolerance under frost stress of 5 °C [37].

Li and Wang [38] reported that the foliar application of different concentrations of salicylic acid greatly increased antioxidant enzyme activities, soluble sugars, proline, and chlorophyll content of grapes under frost stress. Ascorbic acid is a multifunctional metabolite with strong regenerative properties that allows the neutralization of ROS and the reduction of oxidized molecules by ROS in cooperation with glutathione in the Foyer-Halliwell-Asada cycle. Similarly, the important and positive effect of ascorbic acid was reported under drought, salinity, temperature, light stress, and bio stress [39, 40]. Similarly, ascorbic acid has been shown to have a significant and positive effect in the presence of drought, salinity, temperature, light stress, and bio stress [39, 40]. In addition, the external application of ascorbic acid is considered an effective way to increase plants’ tolerance to abiotic stresses [40]. In their research, Mohammadrezakhani and Pakkish [41] stated that grapes, treated with 2 and 4% ascorbic acid, significantly increased the activity of ascorbate peroxidase, superoxide dismutase, catalase, and peroxidase and reduced hydrogen peroxide and electrolyte leakage during cold stress.

Grape (*Vitis vinifera L*.) is one of the most valuable and widely cultivated fruit crops in the world. Iran is the 11th largest producer of grapes in the world with 1.990 million tons of grape production [42]. The grape is a species compatible with the Mediterranean climate and suitable for areas with mild winters and relatively constant temperature changes in autumn and spring [12, 13]. However, grapes are often cultivated outside of these climates in areas with severe winters [43, 44]. Under frost stress, grapes change their soluble sugar concentration, enhance proline synthesis, rectify proteins, and increase antioxidants and phenolic compounds to defend against cold stress [45]. Frost damage can significantly reduce yield or even be a limiting factor of grape growth in cold regions [46]. Rekika et al. [47] showed a significant difference in the survival of primary grape sprouts among the cultivars. The initial sprouts of grapes were much more sensitive to cold than the second sprouts, which were less tolerant than the third sprouts.

This study aims to investigate the effects of some polyamines, salicylic acid, and ascorbic acid on increasing frost tolerance in *Vitis vinifera* L. grape plants. In addition to this investigation of physiological indicators, chlorophyll fluorescence, the antioxidant system network, and frost tolerance were evaluated through the exogenous application of putrescine, salicylic acid, and ascorbic acid in grapes. The results would improve our understanding of the relationship between polyamines, salicylic acid, and ascorbic acid and cold tolerance and provide a new strategy to increase plant frost tolerance.

## Results

### Chlorophyll fluorescence

An analysis of variance (ANOVA) showed that the interactive effects of experimental treatments were significant on Y (II), Fv, maximal fluorescence (Fm), maximum photochemical quantum yield of photosystem II (Fv/Fm)., and Fv/Fo traits at the 1% probability level and on F0 at the 5% probability level. Frost stress increased Y (II) and F0 compared to the control, and the values of Fm, Fv, Fv/Fm, and Fv/Fo decreased in frost stress. Cold stress increased Y (II) and F0 compared to the control, while it decreased Fm, Fv, Fv/Fm, and Fv/Fo. Our results showed that the highest amount of Y (II) was in 0.5 mM salicylic acid at − 3 °C, and the lowest was related to the control. Based on the results, the highest and lowest amounts of Fm were obtained in control and ascorbic acid (200 mg/liter) at − 3 °C, respectively. The highest amount of Fv was observed in putrescine (1 mM) at the control temperature (22 °C), while the lowest value of Fv was observed in untreated plants at − 3 °C. The highest Fv/Fm was obtained from 1 mM putrescine at the control temperature and the lowest value was obtained at − 3 °C without treatment. Our results showed that Fv/Fm was increased by 49, 57, 60, 26, and 34% at − 3 °C with AS (200), Put (5), SA (0.1), Put (1), and SA (0.5) treatments respectively compared to no foliar spraying. The highest and lowest values of F0 were obtained from cold stress without foliar spraying and 200 mg/L ascorbic acid at − 3 °C, respectively. The highest amount of Fv/F0 was obtained from 1 mM putrescine at the control temperature (22 °C), while the lowest value at the untreated temperature was − 3 °C (Table 1).

**Table 1:** Effect of exogenous Put, SA, and AsA pretreatment on Y (II), Fm, Fv, Fv/Fm, F0, Fv/F0 in ‘Giziluzum’ under frost stress

Temperature	Treatment	Concentration	Trait					
			Y (II)	Fm	Fv	Fv/Fm	F0	Fv/F0
-3	Control	0	0.5210 ± 0.004ab	2.514 ± 0.112bc	0.9723 ± 0.125 g	0.3853 ± 0.033e	1.377 ± 0.091a	0.7130 ± 0.124 g
	Putrescine (mM)	1	0.5217 ± 0.002ab	2.095 ± 0.170de	1.009 ± 0.089 g	0.4870 ± 0.071c-e	1.286 ± 0.071ab	0.7850 ± 0.064 fg
		5	0.5347 ± 0.005a	2.167 ± 0.159d	1.302 ± 0.154ef	0.60530.088 ± bc	1.197 ± 0.072b-d	1.099 ± 0.201de
	Salicylic acid (mM)	0.1	0.5363 ± 0.005a	1.995 ± 0.322de	1.215 ± 0.104 fg	0.6183 ± 0.063bc	1.218 ± 0.027bc	0.9967 ± 0.070ef
		0.5	0.5380 ± 0.012a	1.991 ± 0.196de	1.027 ± 0.121 g	0.5170 ± 0.048c-e	1.215 ± 0.020bc	0.8473 ± 0.115 fg
	Ascorbic acid (mgL^−1^)	200	0.5357 ± 0.004a	1.761 ± 0.107e	1.345 ± 0.038ef	0.7653 ± 0.026 a	1.090 ± 0.029e	1.234 ± 0.021c-e
22	Control	0	0.4583 ± 0.016c	3.532 ± 0.124a	1.826 ± 0.099ab	0.5167 ± 0.010c-e	1.213 ± 0.054bc	1.511 ± 0.145b
	Putrescine (mM)	1	0.4627 ± 0.015c	2.586 ± 0.137bc	1.991 ± 0.143a	0.7737 ± 0.085a	1.126 ± 0.011c-e	1.769 ± 0.142a
		5	0.4727 ± 0.009c	3.269 ± 0.124a	1.760 ± 0.144a-c	0.5393 ± 0.051 cd	1.178 ± 0.015c-e	1.494 ± 0.123b
	Salicylic acid (mM)	0.1	0.5123 ± 0.008b	2.755 ± 0.080b	1.542 ± 0.072c-e	0.5600 ± 0.025c	1.182 ± 0.047c-e	1.306 ± 0.068b-d
		0.5	0.5253 ± 0.003ab	2.247 ± 0.123 cd	1.601 ± 0.075b-d	0.7163 ± 0.073ab	1.096 ± 0.025de	1.463 ± 0.101bc
	Ascorbic acid (mgL^−1^)	200	0.5373 ± 0.003a	3.459 ± 0.112a	1.435 ± 0.069d-f	0.4150 ± 0.017de	1.120 ± 0.018c-e	1.282 ± 0.065b-d
	Means of squares	df						
	Temperature		**	**	**	ns	**	**
	Treatments		**	**	ns	**	**	ns
	Temperature×Treatments		**	**	**	**	*	**
	Error		0.000	0.038	0.0018	0.0018	0.003	0.019
	CV		2.05	7.70	9.34	11.56	4.88	11.47

Means with the same letter(s) are not significantly different by Duncan grouping at *P < 0.05*. *, **, ns significant at *p* ≤ 0.05, *p* ≤ 0.01, nonsinificant

### Chlorophyll and carotenoid

**Fig. 1:**
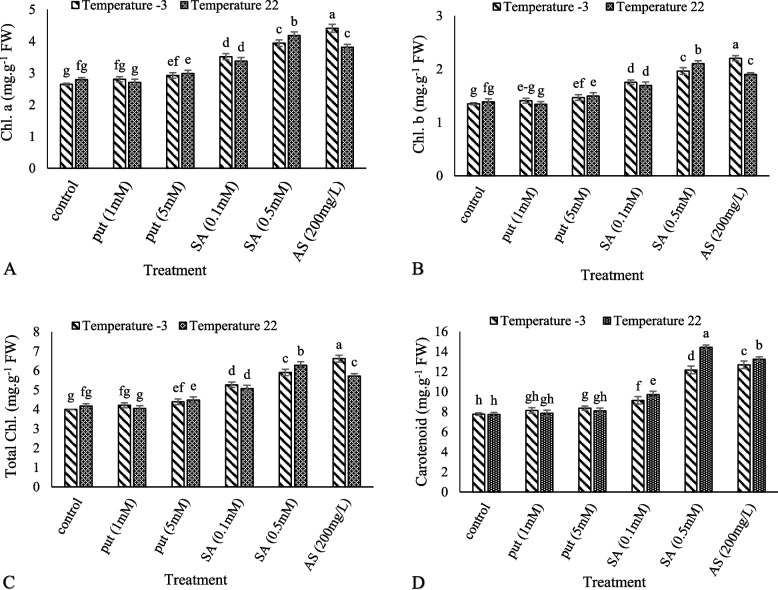
Effect of exogenous Put, SA, and AsA pretreatment on Chl. a (**A**), Chl b (**B**), Total Chl (**C**) and carotenoid (**D**) in ‘Giziluzum’ under frost stress. Different letters are significantly different based on Duncan’s multiple range test (*p* ≤ 0.05)

The highest content of chlorophyll a was obtained from ascorbic acid and the lowest from − 3 °C (Fig. 1A). The highest and lowest chlorophyll b contents were related to ascorbic acid and control at − 3 °C, respectively (Fig. 1B). AS (200), Put (1), Put (5) and SA (0.1) at − 3 °C increased the total chlorophyll content by 45, 63, 4, 8, and 29%, respectively (Fig. 1C). Our results showed that the application of treatments at the control temperature (22 °C) had a better effect on the amount of carotenoid. The highest and lowest amounts of carotenoid were observed at 0.5 mM salicylic acid and without foliar spraying at the control temperature (22 °C) (Fig. 1D).

### Osmolytes and membrane stability

**Fig. 2:**
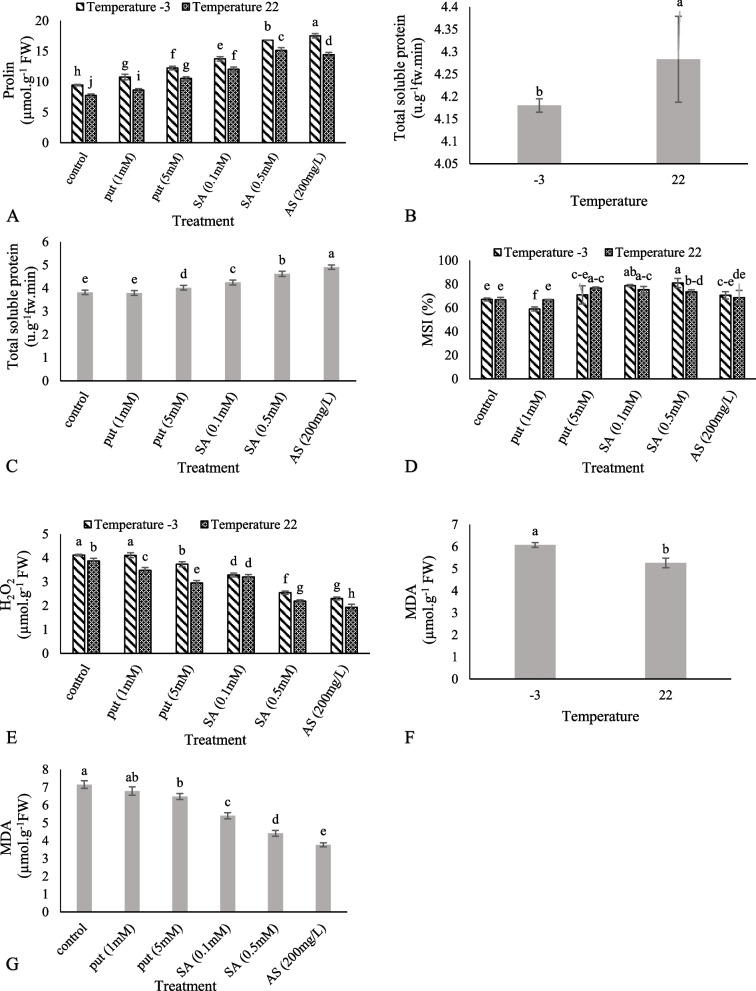
Effect of exogenous Put, SA, and AsA pretreatment on proline content (**A**), total soluble protein (**B** and **C**), MSI (**D**), H_2_O_2_ (**E**) and malondialdehyde (MDA) (**F** and **G**) in ‘Giziluzum’ under frost stress. Different letters are significantly different based on Duncan’s multiple range test (*p* ≤ 0.05)

Frost stress increased proline content. The highest content of proline was obtained from the treatment of 200 mg/L of ascorbic acid at − 3 °C and the lowest value was obtained from the control temperature (22 °C) without treatment (Fig. 2A). Spraying the leaves increased the total soluble protein content in grapes. The results showed that cold stress decreased the content of total soluble protein (Figs. 2B, C). The highest stability index was observed at 0.5 mM salicylic acid and the lowest at 1 mM putrescine at − 3 °C (Fig. 2D). The H_2_O_2_ content increased after applying cold stress. The highest amount was observed at − 3 °C and the lowest amount was observed at 200 mg/L of ascorbic acid at the control temperature (22 °C). The application of Put (1), SA (0.1), Put (5), SA (0.5), and AS (200) decreased the H_2_O_2_ content by 10, 17, 23, 43, and 50%, respectively (Fig. 2E). Malondialdehyde increased in grape leaves upon exposure to − 3 °C (Fig. 2F). The application of Put (1), Put (5), SA (0.1), SA (0.5), and AS (200) decreased malondialdehyde content by 5, 9, 24, 38 and 47% versus the control, respectively (Fig. 2G).

### Antioxidant defense system

**Fig. 3:**
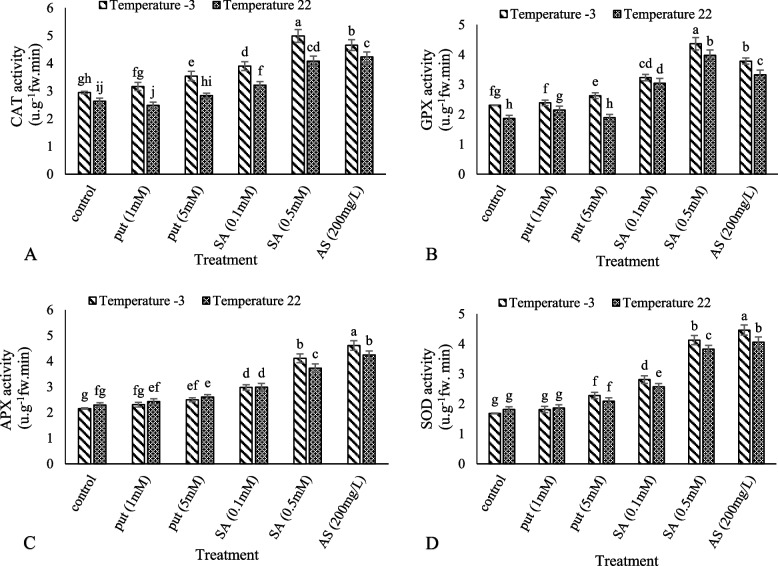
Effect of exogenous Put, SA, and AsA pretreatment on catalase (CAT) (**A**), guaiacol peroxidase (GPX) (**B**), ascorbate peroxidase (APX) (**C**) and superoxide dismutase (SOD) (**D**) contents in ‘Giziluzum’ under frost stress. Different letters are significantly different based on Duncan’s multiple range test (*p* ≤ 0.05)

**Fig. 4:**
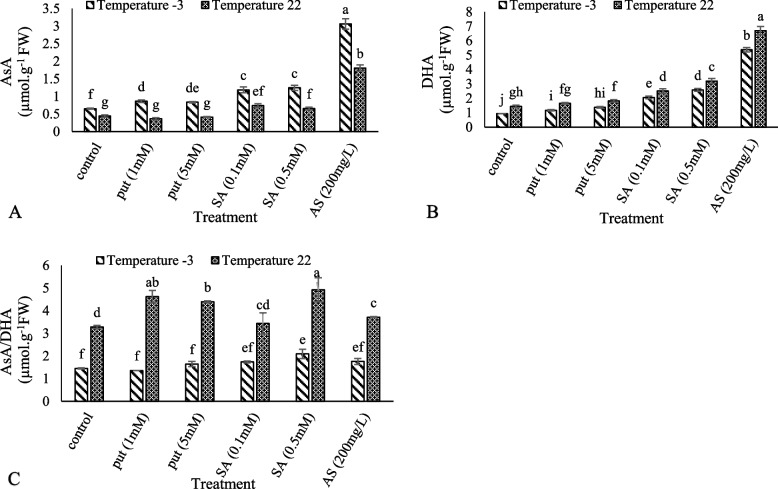
Effect of exogenous Put, SA, and AsA pretreatment on AsA (**A**), DHA (**B**) and AsA/DHA (**C**) in ‘Giziluzum’ under cold stress. Different letters are significantly different based on Duncan’s multiple range test (*p* ≤ 0.05)

The highest CAT activity was obtained from the treatment of 0.5 mM salicylic acid at − 3 °C, while the lowest was observed in 1 mM putrescine at the control temperature (22 °C) (Fig. 3A). The highest and lowest GPX activities were obtained from 0.5 mM salicylic acid at − 3 °C and untreated plants at control temperature (22 °C), respectively (Fig. 3B). The highest APX activity was observed in 200 mg/L of ascorbic acid under cold stress and the lowest was observed in cold stress without foliar spraying (Fig. 3C). The highest and lowest SOD activities were observed in the foliar application of 200 mg/L of ascorbic acid in frost stress and cold stress without foliar spraying, respectively. According to the results, the foliar application of Put (1), Put (5), SA (0.1), SA (0.5), and AS (200) increased SOD activity by 0.07, 0.3, 0.6, 1.4, and 1.6 times in cold stress, respectively (Fig. 3D). The highest amount of AsA was observed in 200 mg/L ascorbic acid at − 3 °C and the lowest amount was observed in 1 mM putrescine at the control temperature (22 °C). The results showed that the application of Put (5), Put (1), SA (0.1), SA (0.5), and AS (200) increased the AsA content by 0.2, 0.3, 0.8, 0.9, and 7.3 fold in frost stress, respectively (Fig. 4A). At the control temperature (22 °C), the treatment with 200 mg/L ascorbic acid showed the highest amount of DHA and the lowest amount was displayed by the untreated plants at − 3 °C (Fig. 4B). The highest ratio of AsA/DHA was observed at the control temperature (22 °C) with 0.5 mM salicylic acid and the lowest value at − 3 °C with 1 mM putrescine (Fig. 4C).

### Correlation matrix and relative expressions

**Fig. 5:**
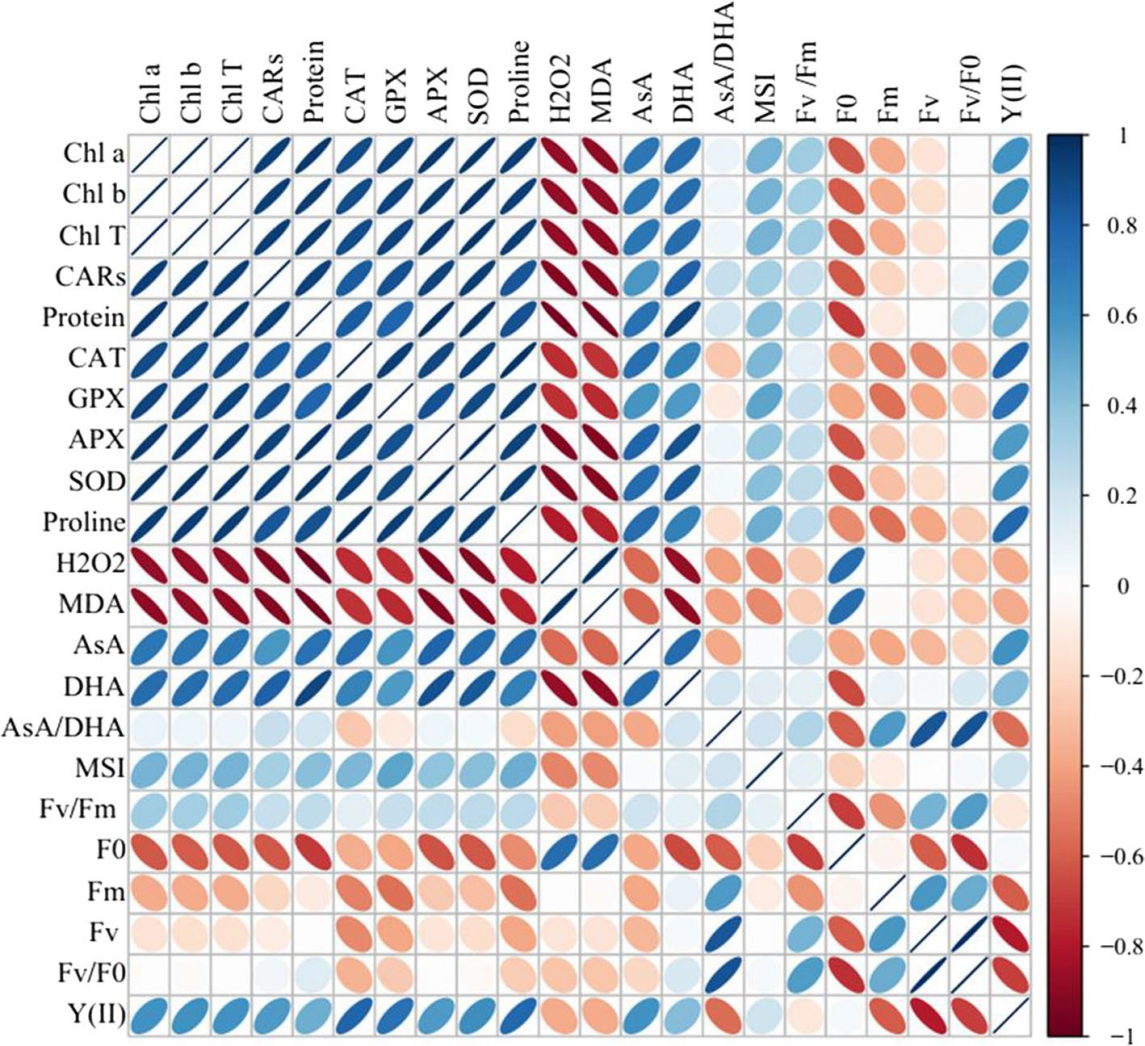
Heat map of Pearson’s correlation analysis. The studied traits included chlorophyll *a* (Chl *a*), chlorophyll *b* (Chl *b*), total chlorophyll (Total Chl), carotenoids (CARs), total soluble proteins content, catalase activity (CAT), guaiacol peroxidase (GPX) activity, ascorbate peroxidase (APX) activity, superoxide dismutase (SOD) activity, proline, H_2_O_2_ content, malondialdehyde (MDA), ascorbate (AsA), dehydroascorbate (DHA), AsA/DHA, (MSI), Fv/Fm, F0, Fm, Fv, Fv/F0, and Y (II)

Pearson’s correlation of photosynthesis pigments, enzymatic and non-enzymatic antioxidants, chlorophyll fluorescence, and biochemical traits is given in Fig. 5, in which a significant positive correlation is observed among photosynthesis pigments, total soluble protein, CAT, GPX, APX, SOD, proline, AsA, DHA, and Y (II). These traits showed a significant negative correlation with H_2_O_2_, MDA, F_0*,*_ and Fm. F_0_ was negatively correlated with AsA/DHA, Fv/Fm, Fv, and Fv/F_0_. As well, Y (II) had a negative correlation with Fm, Fv, and Fv/Fm.

**Fig. 6:**
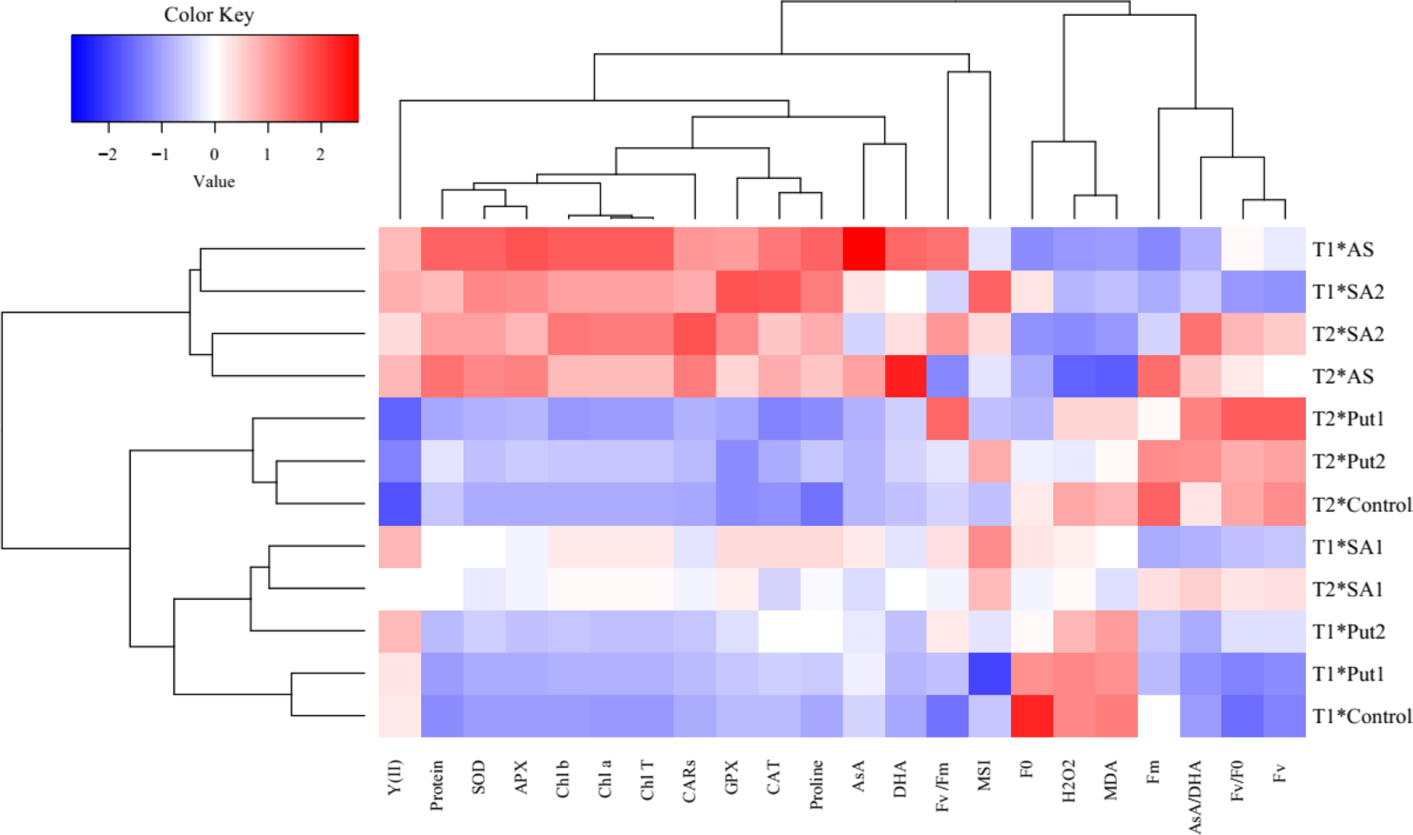
Physiological, biochemical, and chlorophyll fluorescence changes in *Vitis vinifera* cv. ‘Giziluzum’ under frost stress with the treatments of ascorbic acid (AS), salicylic acid (SA), and putrescine (Put). The heat map represents chlorophyll *a* (Chl *a*), chlorophyll *b* (Chl *b*), total chlorophyll (Total Chl), carotenoids (CARs), total soluble proteins content, catalase activity (CAT), guaiacol peroxidase (GPX) activity, ascorbate peroxidase (APX) activity, superoxide dismutase (SOD) activity, proline, H_2_O_2_ content, malondialdehyde (MDA), ascorbate (AsA), dehydroascorbate (DHA), AsA/DHA, (MSI), Fv/Fm, F0, Fm, Fv, Fv/F0, and Y (II)

The heat map (Fig. 6) based on the response of grape plants’ biochemical, enzymatic and non-enzymatic antioxidants, and photosynthesis parameters to the SA, AS, and Put treatments under cold stress showed that enzymatic antioxidant, photosynthesis pigments, proline, AsA, and DHA increased in the plants subjected to the AS and SA. But, these traits decreased under cold stress, while F_0_, MDA, and H_2_O_2_ were enhanced.

Cluster analysis and dendrograms in the heat map (Fig. 6) presented three main groups in the assessed traits of the grape plants under frost stress and foliar applications. Group 1 contained photosynthesis pigments, total soluble protein, AsA, DHA, proline, Y (II), *Fv*/*Fm*, MSI, GPX, CAT, SOD, and APX activity. Group 2 contained other traits including F_0_, MDA, and H_2_O_2_. Finally, group 3 contained Fm, AsA/DHA, Fv/F0, and Fv. In general, the cluster analysis of the heat map for the plants supplemented with the AS, SA, and Put foliar application under cold stress disclosed three main groups. Group 1 contained the grapes subjected to 200 mgL^− 1^ of AS and 0.5 mM of SA under frost stress as well as normal conditions, group 2 contained the plants sprayed with 1 and 5 mM of Put under frost stress, and group 3 included the plants subjected to 0.1 mM of SA under normal conditions and frost stress and the plants supplemented with 1 and 5 mM of Put under normal conditions, and finally the control plants.

## Discussion

The majority of light at its moderate level is used in photochemical activities for photosynthesis, and a small portion of its energy is emitted as fluorescence [48]. In this study, the amount of F0 and Y (II) in the cultivar ‘Giziluzum’ increased due to cold stress. The F0 increase indicates damage to the electron transfer chain of photosystem II due to the decrease in the capacity of quinone A (QA), its incomplete oxidation, and the inactivation of photosystem II [49]. The results also showed that frost stress reduced Fm, Fv, Fv/Fm, and Fv/F0. Researchers argue that the Fm decrease may be related to the decrease in the activity of the water-degrading enzyme and electron transfer cycle in/or around photosystem II [49]. Slowing the entry of D1 protein into the center of photosystem II, frost stress slows down plant recovery, membrane degradation, and chlorophyll oxidation, thereby reducing the Fv/Fm ratio. Frost stress increased F0 and decreased Fm and Fv/Fm in grape cultivars [50]. These findings are consistent with the findings of Gohari et al. in terms of the use of putrescine in grapes under salinity stress [51].

Frost stress can impose negative effects on plant growth and functions. It may alter physiological and biochemical processes in plant cells [8, 52, 53]. In this study, decreases in chlorophyll and carotenoid contents were observed in grapes in response to frost stress. The application of putrescine, salicylic acid, and ascorbic acid treatments reduced the negative effects of frost stress. Cold stress with increased free radicals in chloroplasts injured the cells and reduced membrane permeability [54]. Rapid degradation of chlorophyll is required to prevent cell damage. Frost stress increases ROS in chloroplasts, destroys Chl molecules, and damages the chloroplast membrane system and the photosynthetic reaction center. Salicylic acid, as an ROS detoxifier, may inhibit the activity of free radicals, reduce superoxide radicals, and increase leaf Chl content [55]. The external application of putrescine enhances photosynthetic pigments as reported in some former studies [56, 57]. Gohari et al. [51] reported that the foliar application of putrescine under salinity stress increased chlorophyll content and carotenoids in grapes. Farooq et al. [58] reported that the application of ascorbic acid reduced the effects of water deficit stress and improved the chlorophyll content in four safflower cultivars.

Plants, exposed to environmental stresses, can accumulate various metabolites such as proline to cope with stress conditions [59, 60]. In particular, free proline acts as an osmotic regulator and protector of macromolecules and cell membranes. Free proline also shows antioxidant function [61, 62]. Leaf proline content at frost temperatures increased in the studied grape cultivars and was reported to be different according to the frost tolerance level [63, 64]. This study is consistent with the results of other reports about the increase in proline content under putrescine treatment in grapes exposed to salinity [61] and salicylic acid treatment of wheat plants exposed to frost stress [34]. The foliar application of ascorbic acid significantly increased proline content in flax cultivars under salinity stress [65]. Proteins play a key role in protecting cells from dehydration and damage due to frost stress [66, 67]. In this study, the concentration of total soluble protein in all treated leaves was higher than that in the control. The application of ascorbic acid to the grape cv. ‘Khoshnav’ under drought stress increased total protein content [68]. Haghshenas et al. [69] stated that the foliar application of putrescine and salicylic acid to strawberries increased the total protein content during salinity stress. The mechanisms of cold stress tolerance are related to improving membrane stability, which enables grapes to withstand frost damage.

In this study, we observed that the membrane stability in the grape leaves was increased by frost stress and the foliar application of salicylic acid and ascorbic acid treatments. When both were used, the membrane stability index was higher in the treatment than in stressful conditions. This showed that during the recovery period, the treatments could reduce the frost stress damage to the grape leaf membrane. The foliar application of ascorbic acid and salicylic acid in maize increased undercold stress [70]. According to Zonouri et al. [68], the application of ascorbic acid treatment to grapes cv. ‘Soltana’ and ‘Khoshnav’ exposed to drought stress increased the membrane stability index. The use of salicylic acid in watermelons under frost stress increased the membrane stability index [36]. Salicylic acid reduces the harmful effects of stress factors and significantly reduces electrolyte leakage and maintains the integrity of membranes [35]. The foliar spraying of salicylic acid on grape seedlings has reduced ion leakage and increased frost tolerance by preventing the peroxidation of cell membranes [71].

Plant cell membranes are often the first site of cold stress damage, and MDA is often used as an index of the extent of cell membrane damage [67, 72]. ROS synthesis at high concentrations has destructive effects such as lipid peroxidation, which disrupts membrane integrity and increases MDA content [73]. It was shown that the damage caused by MDA accumulation was alleviated by SA treatment with’White Currant’ and ‘Giziluzum’ under cold stress [74]. Gohari et al. [51] reported that the application of putrescine to the grape cv. ‘Sultana’ decreased MDA content during salinity stress. These results are consistent with the findings of Zonouri et al. [68] for grape cultivars during drought stress with the use of ascorbic acid.

Hydrogen peroxide and active oxygen radicals are produced in natural conditions in very small amounts during normal metabolism in various organs, including chloroplasts, mitochondria, peroxisomes, and wherever an electron transfer chain is found [75]. Increased hydrogen peroxide content in grapes underfrost stress conditions was previously reported [50]. The application of salicylic acid increased the salinity stress tolerance of grapes ‘Sultana’ by reducing H_2_O_2_ content [76]. A decreased level of H_2_O_2_ accumulation was observed in all safflower cultivars due to the external application of ascorbic acid under both control and water stress conditions [58]. The foliar application of putrescine showed that hydrogen peroxide content decreased significantly under drought stress in safflower [77]. H_2_O_2_ acts as a messenger molecule that triggers a cascade of protective reactions in plants against environmental stresses. By increasing hydrogen peroxide, salicylic acid induces the response of various biochemical pathways against stressful effects. Inducible hydrogen peroxide activates the calcium-dependent channel [78].

Grape varieties have different degrees of cold resistance. Differences in cold resistance of grape cultivars are probably a combination of genetic, physiological, biochemical characteristics, environmental conditions, and day length and temperature [11]. Antioxidant systems play a vital role when plants are exposed to frost stress. The main ROS-removing enzymes in plants (CAT, APX, POD, and SOD) are the first defense against the harmful effects of cold stress. In this study, grape leaves, exposed to cold stress, had higher SOD, CAT, GPX, and APX activities than control leaves. Increasing antioxidant capacity is one of the methods used by plants to improve their tolerance to cold stress [67].

Munir et al. [79] reported the increased activity of antioxidant enzymes by applying different concentrations of ascorbic acid in *Ocimum sanctum* L. The SA treatment improved the activity of the CAT enzyme in ‘Giziluzum’ and increased the activity of APX, SOD, and GR in both cultivars under cold stress [35]. Polyamines increase the activity of antioxidant enzymes and non-enzymatic antioxidants [80]. Polyamines also act as direct absorbers of free radicals by binding to antioxidant enzyme molecules. Such results were reported following the external application of PAs including increased activities of antioxidant enzymes (SOD and CAT) which reduced the effects of ROS and membrane damage [81]. Polyamines often play a role in modulating ROS homeostasis in two ways. First, they may disrupt the supply of electrons for ROS generation by preventing the spontaneous oxidation of metals. They may also act directly as antioxidants and scavenge ROS. Secondly, polyamines increase their activity by affecting antioxidant systems. Priming plants with polyamines is effective in inducing tolerance to abiotic stresses such as drought, heat, and cold due to the increase in endogenous polyamines content [82].

ASA, GSH, APX, and GR are important components of the ASA-GSH cycle that plays an important role in inhibiting ROS in organelles, especially chloroplasts [83]. The enzyme ascorbate peroxidase (APX) helps ascorbate in the purification of H_2_O_2_. Ascorbate reacts directly with hydroxyl radicals, superoxide, and singlet oxygen. Ascorbate reduces oxidized forms of alpha-tocopherol. This molecule plays an important role in photosynthesis; therefore, its concentration is high in chloroplasts. In photosynthesis, ascorbate removes hydrogen peroxide produced during the oxygen reduction reaction in photosystem I (Mahler reaction) [84, 85]. The positive effects of external SA on ASA levels under frost stress were documented [86, 87]. In this research, the putrescine, salicylic acid, and ascorbic acid treatments significantly increased ASA/DHA, ASA, and DHA activities compared to the control treatment. These results showed that the treatments eliminated ROS in chloroplasts by improving the ASA-GSH cycle under cold stress. The foliar application of ascorbic acid significantly increased the ascorbic acid content in all safflower cultivars under water stress and non-stress conditions [58].

## Conclusion

Frost stress damaged the grape plants by affecting the photosynthesis system and increasing ROSs. The external application of putrescine, salicylic acid, and ascorbic acid increased the capacity of the antioxidant system, thereby decreasing ROS types, decreasing lipid peroxidation, and increasing membrane stability. Putrescine (5 mM) and salicylic acid (0.1 mM) improved chlorophyll fluorescence indices at − 3 °C. The salicylic acid and ascorbic acid treatments showed the greatest effect on increasing the activity of antioxidant enzymes in non-stress and frost stress conditions. The use of compounds, e.g., ascorbic acid, salicylic acid, and putrescine, strengthens the antioxidant defense system of cells, reduces damage, and stabilizes stable cell conditions under frost stress. Ascorbic acid was more effective than other treatments. in developing cold tolerance in grapes ʻGiziluzum̕ by improving the physiological and biochemical index.

## Methods

The homogeneous one-year-old rooted cuttings of *Vitis vinifera* L. cv. ‘Giziluzum’ were provided by a local nursery in Maragheh, Iran in accordance with the relevant institutional and national guidelines and legislation. Identical cuttings were rooted in pots containing perlite. Then, the plants were transferred to pots containing peat (60%) and perlite (40%) and kept in a greenhouse at day/night temperatures of 25–28/18–20 °C during which they were fed with the Hoagland solution. To carry out the treatments, two-year-old plants were pruned and two branches with two buds were kept, and after 10–15 leaves, the branches were sprayed with foliar spray. The treatments included growth regulators of putrescine, salicylic acid, and ascorbic acid –putrescine at the concentrations of 0, 1, and 5 mM, salicylic acid at concentrations of 0, 0.1, and 0.5 mM, and ascorbic acid at concentrations of 0 and 200 mgL^− 1^ twice and in a 24 h interval. Then, 24 h after the last spraying, the plants were placed in a frost room (for frost treatment) at − 3 °C and control temperature at 22 °C for 3 h. After returning to the greenhouse, the leaves were sampled immediately for the evaluation of membrane stability, enzyme activity, and physiology. For biochemical measurements, young leaves were harvested immediately after the cold, frozen by liquid nitrogen and kept at − 80 °C until analysis.

### Physiological and biochemical assessments

#### Fluorescence

Chlorophyll fluorescence was measured by a fluorometer (model: PAM 2500-WALZ, Germany) from the last fifth leaves in the light. Minimum fluorescence (F0), maximum fluorescence (Fm), and maximum and minimum photochemical quantum efficiencies of photosystem II (Fv/Fm), (Fv/F0), and Y (II) were measured [88].

#### Chlorophyll a and b and total carotenoids

First, 0.5 g of the leaf samples was immersed in dimethyl sulfoxide (DMSO) (3 mL). Then, absorbance was measured at the wavelengths of 480, 649, and 665 nm. According to Wellburn [89], the chlorophyll content was determined in an acetone extract

$$\textrm{Ca}\ \left(\textrm{mg}/\textrm{g}\right)=\left[12.7\textrm{xA}663-2.69\textrm{xA}645\right]\times \textrm{V}/1000\times \textrm{W}\ \left(\textrm{Chlorophyll}\ \textrm{a}\right)$$

$$\textrm{Cb}\ \left(\textrm{mg}/\textrm{g}\right)=\left[22.9\textrm{xA}645-4.86\textrm{xA}663\right]\times \textrm{V}/1000\times \textrm{W}\ \left(\textrm{Chlorophyll}\ \textrm{b}\right)$$

$$\textrm{Ca}+\textrm{b}\ \left(\textrm{mg}/\textrm{g}\right)=\left[8.02\times \textrm{A}663+20.20\textrm{xA}645\right]\times \textrm{V}/1000\times \textrm{W}\ \left(\textrm{Chlorophyll}\ \textrm{a}+\textrm{b}\right)$$

$$\textrm{Where}\ \textrm{V}=\textrm{volume}\ \textrm{of}\ \textrm{the}\ \textrm{extract}\ \left(\textrm{mL}\right);\textrm{W}=\textrm{Weight}\ \textrm{of}\ \textrm{fresh}\ \textrm{leaves}\ \left(\textrm{g}\right).$$

#### Proline

0.2 g of fresh plant material was crushed in a mortar, then 4 ml of 3% sulfosalicylic acid was added to it and placed in ice. Then the samples were centrifuged for 20 minutes at 10000 rpm at 4 °C. 500 μl of ninhydric acid and 500 μl of glacial acetic acid were added to 500 μl of the supernatant solution and mixed. At the same time, 2 ml of standard solutions of 0, 4, 8, 12, 16 and 20 mg/l of proline were poured into glass tubes and 2 ml of ninhydric acid and 2 ml of A liter of glacial acetic acid was added to them and then mixed well. The samples were heated in a hot water bath for 1 hour and finally placed in an ice bath. Then 1 ml of toluene was added to the solution and vortexed for 20 seconds. The reading was done at 520 nm [90].

#### Membrane stability index (MSI)

First, 0.05 g of the leaves was weighted and immersed in 20 cc of deionized water. Then, several samples were kept at 40 °C for 30 minutes. Some other samples were kept at 100 °C for 30 minutes. In the next step, they were placed in an experimental environment to reach room temperature. Electrical conductivity and cell membrane stability were measured with the formula MSI = 1 − (c1/c2) ∗ 100 by EC meter.

#### Measurement of hydrogen peroxide (H_2_O_2_) concentration

To measure hydrogen peroxide, 0.2 g of the leaf sample was homogenized in 2 mL of a 0.1% chloroacetic acid solution (weight-volume) and centrifuged at 12000 rpm for 15 minutes. Then, the reaction complex was obtained by combining 0.5 mL of supernatant, 0.5 mL of 10 mM phosphate buffer at pH 7, and 1 mL of 1 mol potassium iodide. The absorbance of the samples was measured using spectrophotometry at 390 nm. Hydrogen peroxide was obtained using a standard curve [91].

#### Malondialdehyde

The method of Heath and Packer [92] was used to measure the amount of malondialdehyde. For this purpose, 0.2 g of fresh plant leaf sample was homogenized with 1.5 ml of 0.1% trichloroacetic acid (TCA). Then the samples were centrifuged at 4 °C for 10 minutes at 10000 rpm. Then 0.5 ml was removed from the supernatant solution and then 1 ml of thiobarbituric acid (TBA) solution containing 20% trichloroacetic acid was added. The resulting mixture was heated in a hot water bath at a temperature of 95 °C for 30 minutes. To stop the reaction, the vessel containing the heated mixture was placed in an ice bath for 30 minutes. After cooling the mixture, centrifugation was performed at 10000 rpm for 10 minutes. Finally, the absorbance of the mixture was read by a spectrophotometer at 532 nm and 600 nm. In calculating the value of MDA, the extinction coefficient cm-1 mM-1155 was also taken into account. Finally, MDA was calculated in terms of nmol/g FW using the following formula.

$$\textrm{MDA}=\left[\left(532\;\textrm{nm}-600\;\textrm{nm}\right)\times 20\right]/155\times 100$$

#### Antioxidant enzymes assay

To measure total soluble protein, catalase, and guaiacol peroxidase, first, 0.5 g of the plant sample (leaf) was homogenized in liquid nitrogen. Then, 2 mL of phosphate buffer (pH = 7.5) containing EDTA (0.5 mol) was added. The samples were incubated at 4 °C for 15 minutes and before being centrifuged at 15000 rpm. Due to the instability and very low half-life of ascorbate peroxidase in ex-vivo conditions, polyvinylpyrrolidone 5% and ascorbate (2 mL) were added to the respected enzyme solution to preserve its structure [93].

#### Total soluble protein concentration

To measure protein, 5 mL of Bradford reagent was added to 0.1 mL of the protein extract of each sample. Then, it was vortexed for 20 minutes, and adsorption at 595 nm was recorded. In this method, standard curves obtained from the determined concentrations of standard proteins were used to measure the amount of protein [94].

#### Catalase enzyme activity

The catalase (CAT) enzyme activity was investigated given the reduction of hydrogen peroxide at 240 nm. The reaction mixture consisted of 50 mM of phosphate buffer (pH = 7) and 15 mM of hydrogen peroxide. The reaction was started by adding 100 mL of the enzyme extract to the final volume, which was 3 mL. Adsorption changes were recorded at 240 nm for 3 min. The enzyme activity was then expressed as changes in adsorption per minute per milligram of protein [95].

#### Ascorbate peroxidase enzyme activity

To measure the activity of the ascorbate peroxidase enzyme, the reaction mixture consisted of 250 mM of phosphate buffer (pH =7), 1.2 mM of hydrogen peroxide, 0.5 mM of ascorbic acid, and 1.0 mM of EDTA. The enzymatic activity was initiated by adding hydrogen peroxide to the mixture. The light absorption, which decreased due to ascorbic acid peroxidation, was read for 2 minutes by a spectrophotometer at 290 nm. The changes in absorption per minute per milligram of protein were used to calculate the enzyme activity [96].

#### Guaiacol peroxidase activity

To measure the activity of the guaiacol peroxidase (GPX) enzyme, the reaction medium consisted of 25 mM of potassium phosphate buffer (pH = 6.8), 40 mM of hydrogen peroxide, and 20 mM of guaiacol. The reaction was started by adding 100 μl of enzyme extract to the final volume; i.e., 3 mL. The increased adsorption was recorded by tetragayacol formation at 470 nm for 3 minutes. The enzyme activity was then expressed as the change in absorption per minute per milligram of protein per minute [96].

#### Superoxide dismutase (SOD) activity

To measure the activity of superoxide dismutase enzyme, 1500 μl of 100 mM phosphate buffer, 200 μl of 0.2 mM methionine, 100 μl of EDTA (3 mM), 900 μl of distilled water and 100 μl of sodium carbonate (NaCO3) 1.5 Molar and 100 μl of riboflavin were mixed together, and at the end, 50 μl of the enzyme sample was added to each test tube. Then the test tubes were placed at a distance of 30 cm from the light source for 15 minutes. After that, they were kept in complete darkness for 15 minutes and at the end of the work, the absorption changes of the samples were read using a spectrophotometer at 560 nm [97].

#### Reduced and oxide ascorbate

The antioxidant activity of AsA was measured using0.2 g of the plant sample and 10% metaphosphoric acid, 150 mM phosphate buffer, TCA (10%), 44% phosphoric acid, 4% biperiden, and iron chloride (FeCl3). After vortexing the mixture of the samples, the absorption changes of the samples were read by a spectrophotometer at 525 nm. The enzyme activity was calculated by the ascorbate standard curve. So, 0.2 g of the plant sample was used to measure ascorbate enzyme activity. Then, 10% metaphosphoric acid was used for homogenization. The reaction complex included the extract, 150 mM phosphate buffer, and 10 mM dithiothreitol. Finally, TCA (10%), phosphoric acid 44%, bipiperidyl 4%, and iron chloride (FeCl3) were added. Then, the samples were kept at 37 °C for 1 hour. Finally, the absorption changes of the samples were read by a spectrophotometer at 525 nm. The enzyme activity was calculated by the ascorbate standard curve [98].

#### Statistical analysis

The research was analyzed based on a factorial experiment in a completely randomized design using MSTATC statistical software (version 2.10).. The figures were created using heatmap graph analysis, data correlation analysis, and Excel (2016). The means of the data were compared with Duncan’s multiple range tests at the probability levels of 5 and 1%. Tables and charts were drawn using Office software.

## Data Availability

The data that support the findings of this study are available from the corresponding author upon reasonable request.

## Abbreviations

SOD: Superoxide dismutase
CAT: Catalase
APX: Ascorbate Peroxidase
MDA: Malondialdehyde
GPX: Guaiacol peroxidase
GR: Glutathione reductase
AsA: Ascorbate
SA: (Salicylic acid)
